# Architecting Functional Polymers: Advances in Modular Synthesis, Responsive Design, and Multifaceted Applications

**DOI:** 10.3390/polym18030334

**Published:** 2026-01-26

**Authors:** Akhil Sharma, Monu Sharma, Sonu Sharma, Vikas Sharma, Shivika Sharma, Iyyakkannu Sivanesan

**Affiliations:** 1 School of Bioengineering and Biosciences, Lovely Professional University, Jalandhar 144411, Punjab, India; sharmaakhil177@gmail.com (A.S.); biotech_vikas@rediffmail.com (V.S.); shivikasharma25@gmail.com (S.S.); 2 Baba Nahar Biotech and Research, Bilaspur 174001, Himachal Pradesh, India; monusharma311998@gmail.com (M.S.); sharmasonu70462@gmail.com (S.S.); 3 Department of Environmental Health Science, Human and Eco Care Center, Konkuk University, Hwayang-dong, Gwangjin-gu, Seoul 05029, Republic of Korea

**Keywords:** architected polymer systems, modular polymer synthesis, stimuli-responsive polymers, controlled polymerization, post-polymerization modification, multifunctional polymers, adaptive polymer architectures

## Abstract

The recent development in polymer science has gone beyond the traditional linear and randomly functionalizable macromolecules to the architected polymer systems, which integrate modular synthesis and dynamic responsiveness. Although the literature related to polymer synthesis and stimuli-responsive materials and applications is widely discussed, it is common to review the aspects independently, restricting a complete picture of how architectural modularity controls adaptive performance. This gap is filled in this review with an integrated framework of relating modular polymer synthesis, stimuli-responsive design, and application-oriented functionality in a single coherent design philosophy. The scientific novelty of this review is that the focus on modular polymers is not only on synthetic constructs, but is a programmable functional scaffold where the structural precision is the direct determinant of responsiveness, multifunctionality, and performance. Controlled polymerization and post-polymerization modification regimes are mentioned to be tools that allow precise positioning of functional modules, and this allows polymers to respond in predictable ways to environmental stimuli like pH, temperature, light, redox conditions, etc. In addition, the review identifies the role of a synergistic combination of various responsive modules in the emergence of behaviours that would not be reached in conventional polymer systems. This review offers a coherent viewpoint on the future of functional polymers of the next generation by bringing together synthetic approaches to nano-responsive behaviour and real-world technologies, such as drug delivery, self-healing surfaces, adaptive surfaces, and biosensing surfaces. The framework in the present paper provides a logical route towards the development of environmentally friendly, multifunctional, and adjustable polymer structures.

## 1. Introduction

### 1.1. Evolution of Polymer Architectures: From Conventional Linear Systems to Modular and Architected Polymers

Polymer science has experienced a radical change in the past few decades, shifting towards the production of simple linear macromolecules, towards the controlled formation of architected polymer system structures, and towards the functional complexity of extraordinary complexity [1]. Homopolymers and random copolymers dominated the early polymer architecture, whose properties were mainly determined by composition, chain length, and degree of polymerization. Despite the enabling role of these materials in the evolution of polymer studies, their tunability in functionality was still not very high, and the capacity to finely tune properties was still restricted by the inherent solidity of the conventional polymerization methods [2]. Branched, star-shaped, dendritic, networked, and block copolymer systems raised the paradigm of polymer structures by offering new freedom in the manipulation of morphology, self-assembly, and mechanical behaviour [3]. Even these advanced designs, however, were more commonly assembled by a process that was neither as modular nor as programmable as would be required to create finely tuned, multifunctional materials [4].

In recent years, further development of controlled polymerization, monomer design, and post-polymerization modification has re-established the design of polymers through the construction of polymers as modular systems, not in the form of monolithic chain polymers [5]. In modular polymer systems, functional units are programmable, individual building blocks, such as monomers, functional segments, or responsive modules, which can be combined in an extremely controlled and predictable manner [6]. This is a modular paradigm that enables the researcher to directly encode the structural order, chemical heterogeneity, and adaptive behaviour in the polymer backbone or the side-chain structure. Consequently, the correlation of structure and properties is more readily available, and the design of multifunctional materials is highly systematic and not based on intuition [7]. This move towards modularity is part of a larger movement in the whole of materials science: a shift in passive materials to an active system that has active interaction with its surroundings [8]. This type of evolution resembles the complexity in biological polymers, in which folding, responsiveness, and functional specificity are controlled by modular functional units. Synthetic polymer design has started to mimic the property of nature, whereby, through an architectural philosophy, several functional motifs were able to be incorporated within a single macromolecular architecture, allowing unprecedented behaviour of the molecular structure and material performance [9].

#### Conceptual Framework of Modular Synthesis and Modular Polymer Architectures

In polymer science, modular synthesis is a design approach where polymers are built up of discrete, functional building units instead of being oriented as uniform chains with randomly distributed functional groups. In this strategy, the individual monomers, blocks, or functional segments are considered as separate modules, which are programmed to carry out a particular structural or functional task. These modules may be synthetically assembled, swapped, or reorganized in vivo or in vitro and permit the polymers to be assembled with a great deal of architectural and functional precision [10].

Unlike the traditional method of synthesis of a polymer, where bulk composition and overall molecular weight largely determine polymer characteristics, the modular synthesis of polymers permits the control of sequence, spatial distribution, and other properties of functional modules with intentionality. The modules still maintain their chemical identity and act cooperatively towards the overall behaviour of the material. Such a notion is analogous to biological macromolecules in which different modules carry out a specialized function yet are part of a single functional unit [11].

The structural result of this synthetic philosophy is called modular polymer structures. They report polymer systems where functional modules are arranged into well-structured polymer structures in the form of block copolymers, segmented networks, grafted chains, or multi-module macromolecules. Modular architectures are characterized not only by structural complexity but also by the ability to programme structure–property relationships, with material performance being predicted and optimized by simply changing individual modules without having to redesign the whole polymer structure.

Notably, modular synthesis and modular architectures are inseparable. Modular synthesis is the chemical toolkit that is used to construct modular architectures, whereas the architecture is used to regulate the interaction of the various modules, their self-assembly, and responses to external stimuli [12]. Such a relationship enables polymers to display the following behaviours: multi-stimuli responsiveness, synergistic multifunctionality, and reversible adaptation, which would be hard to accomplish when the polymers are linear or randomly functionalized.

### 1.2. The Modular Synthesis with Its Responsive Design: Transformative Significance

One of the most radical changes in modern research in polymers is the intersection of modular synthetic strategies and stimuli-responsive design [13]. Responsive polymers are exceptional polymers that can change their physical or chemical properties as a result of certain environmental responses, including pH, temperature, ionic strength, light, redox potential, or mechanical stress [14]. When these responsive components are integrated into a modular structure, they provide very fine-grained, reversible, and contextualized changes in material behaviour. Such a combination makes it possible to develop intelligent polymer systems that can be self-regulated, autonomously adapt to the environment, and maintain multi-stage functional activity [15].

Unlike conventional polymers, where the properties of the polymers are fixed once they are prepared, modular-responsive architectures are dynamic systems that can sense and respond to environmental cues [16]. This property greatly broadens their applications in a considerable variety of applications, including drug delivery carrier systems that liberate therapeutic agents only upon specified physiological signals and self-healing materials that can restore the structural integrity of mechanical damage [17]. Moreover, modularity offers the capability to incorporate a number of responsive units into one polymer, providing synergistic interactions and multifunctional behaviour that could not otherwise be achieved with traditional linear or random architecture [18].

The potential of modular synthesis combined with responsive design is also transformative, especially when it is boosted by the development in precision chemistry. Architected polymers can be synthesized using controlled polymerization methods, including ATRP, RAFT, and ROMP, to achieve narrow dispersities, block sequence control, and a very precise functional group position [19]. Post-polymerization alterations present further possibilities of incorporating a functional module in space and chemical accuracy. These approaches together offer the synthetic backbone on which highly orchestrated responsive systems can be constructed, whereby each functional element has a particular function in the larger architecture [20]. Such a change is not confined to chemical precision only. Modular synthesis is a combinatorial approach that enables researchers to adapt the functionality to the needs of a particular application [21]. As an illustration, the addition of redox-responsive moieties and hydrophilic blocks can be used to create drug delivery systems that react to intracellular glutathione gradients. Similarly, the interactions of photoreactive units with mechanically strong backbone geometries generate adaptive surfaces, i.e., surfaces that become hard or soft under regulated light [22]. In this way, with the addition of stimuli-responsive capabilities, modular synthesis provides a system where more complex multifunctional and application-specific polymers can be rationally designed [5].

### 1.3. Bridging Synthetic Strategies, Responsive Design, and Application-Oriented Innovation

Although significant advancements have been made in the area, the most common issue is how to close the gap between the synthesis of polymers, responsive behaviour, and end-use functionality [23]. Synthetic chemistry innovations are not necessarily converted into viable materials unless, through the prism of application relevance, they are screened. Equally, applications that demand adaptive behaviour are impossible to achieve without underlying systems that can control chemical and architectural elements in a precise manner [24]. Thus, the need to have a unifying system that combines the synthetic methodology with the responsive design and application requirements has become critical to the progressive future of functional polymers [25]. It is at this important crossroad that the current review highlights how modular synthetic approaches can be used to allow architectures to be programmed, the responsiveness of domains to provide adaptability and multifunctionality, and how the combination of these properties can be used to develop superior technologies across biomedicine, energy systems, and the environment [26]. The modular approach offers a special chance to project structural parameters to responsive behaviour and eventually to actual performance. An example is that the ability to position hydrophobic, hydrophilic, degradable, or sensing units with a specified position in the polymer chain allows close control over assembly pathways, degradation, mechanical behaviour, and responsiveness [27]. These properties have direct implications on the design of drug delivery vectors, self-healing elastomers, adaptive protective coating, and chemical sensors, and graphically show the necessity of having a holistic relationship between synthesis and usage [28].

**Figure 1:**
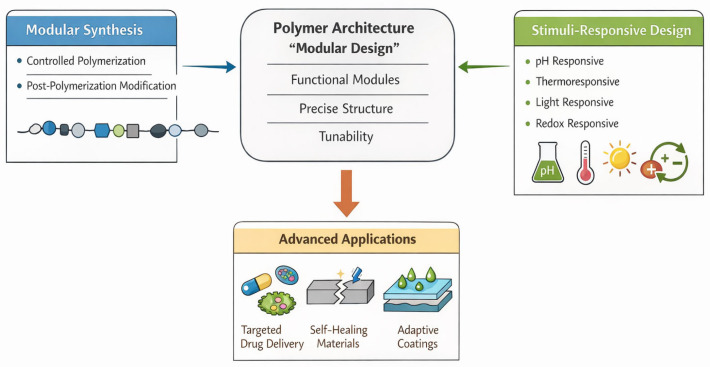
Conceptual map of architected modular polymer systems showing how modular strategies of synthesis, polymer architecture, and stimulus-responsive design merge. The figure points out that this has been made possible by controlled structural organization, allowing tunable properties and additional applications like targeted drug delivery, self-healing materials, and adaptive coatings.

The key to these areas is also to realize the role of efficiency, scalability, and sustainability in the current polymer design. The growing trend toward ending up with environmentally sound production procedures brings in the necessity of synthetic approaches that reduce the amount of waste, shorten reaction time, and allow recycling or biodegradation [29]. These goals are inherently supported by modular systems because synthesis routes can be much more predictable, so that trial-and-error modification is not needed. In addition, the emergence of computational and machine learning software offers a novel interface through which synthetic design is connected to functional prediction [30] (Table 1) (Figure 1).

### 1.4. Scope and Novelty of the Current Review

The current review is comprehensive and integrative and relates techniques of modular synthesis, responsive design of polymers, and iso-topological application landscapes [36]. This article, unlike the traditional reviews that have focused on discussing the themes as separate issues, has made an effort to point out how these themes are interdependent and how they can be effective when they are considered to be parts of a holistic design framework. This is the novelty in this work: to describe modular polymer architectures not only as a synthesis but also as functional scaffolds with highly tunable, adaptive, and application-oriented performance [37]. This review will describe a vision of the future of polymer structures by exploring new trends in controlled polymerization, multifunctional module integration, stimuli-responsive systems, and computational design, aiming to create generalizable, multifunctional, and sustainable polymer structures [38].

This is an integrative strategy that will point out the role of structural modularity and responsive behaviour in transforming the boundaries of polymer science. In this discussion, this review highlights how architected polymers can be used as the backbone to future technologies in biomedicine, energy storage, environmental remediation, and more [39]. The point of view provided here not only summarizes the current progress but also describes the emerging issues and potentials, providing a platform to further development and innovation in the creation of highly functional, environmentally friendly, and universally adaptable polymer systems [40].

## 2. Modular Architectures in Polymer Design

### 2.1. Principles of Modular Polymer Assembly

A major conceptual shift in the synthesis of polymers with the rise in modular polymer assembly is the consideration that macromolecules are formed by designed groupings of functional monomers, instead of the same chains formed by polymerization in one step [41]. The fundamental principles of this strategy are the rational choice of monomeric units and the controlled positioning of functional groups, which, in general, determine the structure and behaviour of the polymer. In contrast to conventional methods, in which the properties of the polymer are mostly due to the composition and sequence distribution of a restricted repertoire of monomers, modular assembly allows the intentional incorporation of chemically diverse building blocks in manners that bring about plied functionality, spatial structure, and specific reactivity [42]. The intentionality of this architecture is similar to that of the hierarchical construction of the biological macromolecules, in which different modules perform their specific biochemical functions, but at the same time have cooperative interactions with the larger structure [43].

The range of monomer structures by which monomers can be assembled to form a structure remains open to increase with the range of synthetic chemistry expanding the repertoire of monomers with orthogonal reactive handles and stimuli-responsive motifs, as well as structurally rigid or flexible components. Individual monomers or functional units are chosen despite their inherent qualities, as well as their suitability to the rest of the architectural blueprint [6]. The functional groups can be arranged to form pre-established spatial patterns that affect either chain folding, module segregation, or intramolecular interactions. This is important in designing polymers with the potential of an environmental sense, shape adjustment, or specific molecular recognition [44]. Researchers can design polymers that react selectively to certain stimuli, degrade under programmed situations, or react selectively towards biological or chemical targets by selecting monomeric units with defined reactive pathways, or with the potential to interact, or not, with specific biological or chemical targets [45].

The modularity of assembly also allows the structure–property relationships to be tailored with a more precise level of control previously unattainable with the traditional random copolymerization [46]. Modular assembly allows the study of the effect on individual parts of the polymer to understand the influence of individual chemical modules on mechanical rigidity, thermal cycling, solubility characteristics, or intermolecular interactions. This relationship can be used to design systems where the small changes in the choice of the monomers can be converted into predictable and finely tuned changes in macroscopic behaviour [47]. The control of these relationships is important, not only to maximize the performance, but also to learn the basic principles according to which polymer behaviour is understood. The controllable interaction between local chemistry and global behaviour in modular architectures has become a useful design tool enabling researchers to create materials that meet critical performance demands in applications like biomedical engineering, functional coatings, and advanced energy materials [48].

The use of modular polymer assembly also has the advantages of synthetic methods that reduce cross-reactivity and enable sequential or hierarchical assembly [49]. Directed polymerization processes enable one to insert segments in a specific sequence in a way that maintains the integrity of the reactive groups that will be used later during assembly. This is supplemented by post-polymerization modification strategies that allow functionalization of individual locations on the polymer chain after synthesis of the backbone [31]. The pre-polymerization design and post-polymerization accuracy lead to architectures whereby separate modules maintain their identity whilst providing the cooperative characteristics of the material [50].

### 2.2. Multifunctionality Through Modular Integration

The real strength of modular polymer design is seen when several functional modules are incorporated into one macromolecule, allowing the formation of complex multifunctional behaviour that is not possible with even linear or homogeneously functionalised polymers [51]. In modular integration, each functional unit is selected due to its ability to perform a particular task, e.g., respond to a stimulus, bind to some target, mediate self-assembly, or provide mechanical reinforcement, and be compatible with other units in the architecture [52]. These modules, when combined in a coherent structure, mutually respond to create a more versatile, responsive, and performance-oriented polymer structure.

The multifunctionality of modular polymers is not simply the addition of the functional groups, but rather the interaction between modules that can affect the behaviour of one another [53]. Indicatively, the delivery of self-assembled morphologies, such as encapsulation, transportation, or the release of specific molecules, can be achieved by including hydrophilic and hydrophobic segments in a rational design. By incorporating reactive sites next to mechanically strong components, systems that can heal, stiffen, or soften depending on the environmental conditions can be realized [54]. These synergistic interactions give it a certain degree of adjustability, enabling the polymer to execute more complicated tasks, including targeted therapeutic delivery, chemical sensing, environmental remediation, or dynamic modulation of surfaces [55].

The strategic arrangement of numerous functional components also allows the polymer to act under a variety of stimuli at the same time or in a sequence, increasing the potential applications of the polymer [56]. An example is that a polymer can have a thermal-responsive block that can change the chain folding, a pH-sensitive group that can change solubility, and a redox-sensitive group that can trigger chemical reactions in an oxidative or reducing environment. When such units are brought to one architecture, the polymer can then coordinate multi-stage activities, including sensing, structural transition, and controlled chemical release [57]. The task of such systems is an extremely calculated and complex process because the coordination between modules depends on the spatial distribution and the chemical compatibility of modular components [58].

The other benefit of modular multifunctionality is that it can be used to produce polymers with adjustable performance profiles that are customizable to suit the requirements of various application environments. In biomedical applications, the polymer can be designed to be biocompatible and also retain the ability to respond to a particular physiological stimulus, improve cellular interactions, or disintegrate innocuously upon effecting its task [55]. Modularity can be used in energy materials to integrate conductive, insulating, and charge-storage units into the same structure and, thus, convert them into flexible electronics, batteries, or electrocatalytics. In the case of environmental uses, modular polymers can be used with pollutant-binding motifs in combination with responsive modules that can be used to regenerate or release captured species [59]. The above-mentioned examples highlight the broadness of the potential of modular integration to facilitate materials’ response to the complex and variable demands of operation [60].

The introduction of several modules also contributes to the polymer performance being strengthened synergistically [61]. Functional groups that can form hydrogen bonding, π-- interactions, or reversible covalent bonding can be incorporated in order to have materials with structural reinforcement or better self-assembly behaviour [62]. With a suitable balance of these interactions, researchers are able to create polymers that are both mechanically robust and able to adapt to the environment or reconfigure themselves. This kind of synergy is necessary in the development of high-level materials that have to endure varying conditions without losing or deteriorating functional capability [63].

### 2.3. Structure–Property Control in Modular Polymers

The ability to impose specific control over the structure–property relationship to enable the engineering of materials with highly specific mechanical, thermal, optical, and chemical properties is one of the characteristic strengths of modular polymer design [64]. In conventional polymer systems, it is difficult to obtain such precision due to the unpredictability of the monomer distribution and functional group distribution, and the characteristics are not the result of an architectural structure but rather the result of bulk composition [65]. The limitations are solved in modular architectures using planned positioning of functional modules, sequence distribution, and variation in segment length, composition, and connectivity. This manipulation of mechanical properties can be attributed to the large extent to which the stiffness of the chains, the density of crosslinks, segmental mobility, and supramolecular interactions can be controlled using a modular design [66]. The addition of rigid units to the selected areas enhances the elastic modulus and resistance against deformation, and the addition of flexible or elastomeric segments offers energy dissipation and elasticity. By composing these segments in a structured architecture, investigators can adjust the response of the mechanical behaviour of a continuum between brittle and ductile behaviour. This type of structural customization is especially useful with biomedical implants, artificial tissues, and flexible electronics, where mechanical matching between biological/device and device environments is a necessity [67].

It is also possible to control thermal properties equally well in modular polymers. The polymer can be tailored to be stable at a given set of operating conditions as well as to experience controlled transitions in the presence of desired stimuli by choosing blocks with known glass transition temperatures, melting points, or profiles of thermal degradation [68]. This plays a vital role in applications that require the use of thermal actuation, phase change materials, and high-performance coatings. The modular method allows for building architectures in which thermal behaviour is not globally homogeneous but will be locally heterogeneous in the polymer, with the capability of complex thermal responses and spatially heterogeneous functionality [69]. The other area of structural control is optical properties. The polymer backbone or side chains can have chromophoric or photosensitive units to control light absorption, emission, or refractive index. Modular assembly is performed such that these optical elements are installed in a format that facilitates the effective communication of modules, minimizes quenching effects, or controls energy transfer. This precision is essential in the design of optical sensors, photonic materials, and the actuators, which are driven by light [70].

**Figure 2:**
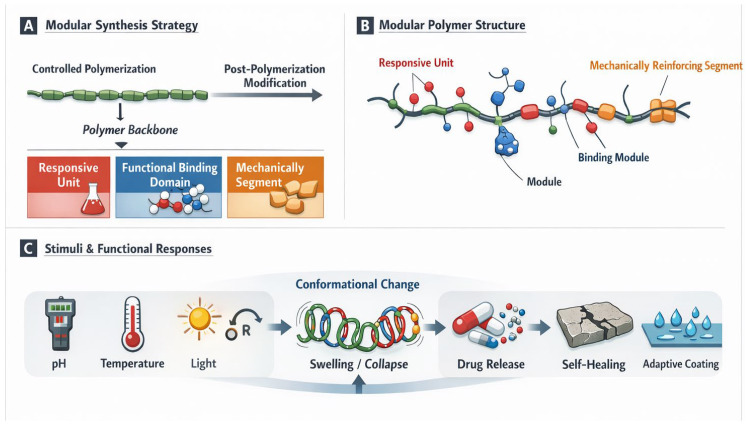
Schematic illustration of modular polymer architecture showing the stepwise synthesis strategy, spatial arrangement of functional modules along the polymer chain, and the resulting stimulus-responsive functional outcomes.

Modularity can also be used to control chemical reactivity and stability. Polymers can have selective chemical behaviour by having reactive motifs and protective or stabilizing modules, e.g., targeted degradation, control of bond cleavage, or catalysis [71]. This degree of control enables the material to be designed to meet the needs of both an environmentally demanding durability and a controlled reactivity, i.e., biomedical scaffolds or environmental cleanup systems, and also advanced coatings technology [72]. The general benefit of modular structure–property control is that it produces application-specific materials, the behaviour of which is not a result of trial-and-error synthesis but must be specified by design. This matches the development of polymers with the needs of precision engineering, and modular architectures are essential in the next generation of functional materials [73]. To enable an intuitive interpretation of this idea, the modular polymer architecture is schematically represented by accelerating the hierarchical synthesis, spatial localization of functional modules, and the attained structure–property interrelations. The schematic underscores how it is possible to predict adaptive behaviour of a system by controlled positioning of the responsive, functional, and reinforcing modules within a single polymer framework due to the influence of external stimuli (Figure 2).

#### Role of Nanomaterials and Carbon Nanostructures in Smart Polymer Composites

Nanomaterials incorporated into polymer structures have become a promising approach to addressing the inherent weaknesses of polymers, particularly their low electrical and thermal conductivity. As part of the modular polymer design, nanomaterials serve as individual functional units that can be strategically inserted throughout polymer matrices to provide a high level of physicochemical functionality without compromising structural rigidity. Of the many nanomaterials, carbon-based nanostructures, carbon nanotubes (CNTs), graphene, graphene oxide, and carbon nanofibers have attracted special interest because of their outstanding electrical conductivity, thermal transport capacity, and mechanical strength [74].

Carbon nanostructures are embedded into polymer matrices to create percolating conductive networks, which make a major contribution to improving charge transport and heat dissipation. Most of these enhancements are greatly influenced by the dispersion condition, interfacial relations, and spatial arrangement of nanofillers in the polymer structure. Controlled polymerization and modular synthesis methods provide a specific arrangement of functional groups that facilitates good interfacial bonding of polymers and carbon nanostructures, thus minimizing thermal resistance at the interfaces and enhancing electron flow across the interface of the composite. Such a controlled incorporation enables conductivity to be enhanced by rather low filler loadings, without sacrificing polymer processibility and flexibility [75].

In intelligent composite, the synergistic effect between stimulus-responsive polymeric matrices and conductive nanofillers can allow the adaptive electrical and thermal behaviour. Indicatively, a thermo-responsive or strain-sensitive polymer with CNTs or graphene can be used to have the electrical conductivity change reversibly in relation to temperature, deformation, or other environmental factors. This behaviour has found special uses in flexible electronics, wearable sensor applications, electromagnetic interference shielding, and self-monitoring structural materials [76]. In the same line, graphene-reinforced polymer composites and CNL-reinforced polymer composites have proven significant enhancement in thermal conductivity and should be used in the thermal management of electronic equipment and energy storage systems.

The multifunctionality is also aided by carbon nanostructures, which can increase the mechanical strength, electrical conductivity, and thermal stability. Such nanofillers can be considered as functional reinforcement modules that can be used interactively with responsive polymer segments in the modular polymer approach. Such a design approach requires a hierarchy so that conductivity enhancement will not be a separate property but rather a part of the adaptive response of the material. Modular polymers produced by nanocomposites can, therefore, be viewed as an important class of smart materials; the intersection of structural programmability, responsiveness, and improved transport properties enlarges the usage space of architected polymer systems [77].

## 3. Advances in Controlled and Sustainable Polymer Synthesis

### 3.1. Controlled Polymerization Techniques

A key breakthrough in the rational design of architected polymer systems is the development of controlled polymerization methods that, with a high level of precision, allow control of the length of chains, their dispersity, sequence distribution, and topological complexity [78]. Such methods have offered the synthetic basis by which modular polymer structures can be built, in which each cleft performs a known structural or functional purpose. The standard polymerization techniques seldom permit this kind of precision since they produce polymers that have a wide range of molecular weights and no control over the incorporation of the monomers [79]. Conversely, polymerization mechanisms involving controlled processes, especially those based on reversible processes of deactivation, enable the chemist to control chain propagation with near-molecular precision. This provides uniformity as well as reproducibility, guaranteeing the translation of modular polymer architectures to reliable functional material [80].

The reversible deactivation radical polymerization techniques have been created as a mandatory tool in the preparation of well-defined macromolecular structures, among the methods of controlled polymerization [27]. These are the atom transfer radical polymerization, reversible addition and fragmentation chain transfer polymerization, and the nitroxide-mediated polymerization. Both techniques come to control by temporarily silencing active ends of chains, thus avoiding uncontrolled propagation and enabling monomers to be inserted with a predictable kinetics. Through this control, it is now possible to form block copolymers, gradient sequences, star structures, and multisegmented polymers where the geometry of the functional module is well-coordinated [81]. The capability to decide on the precise position of responsive, catalytic, or structurally rigid units has great benefit in the design of modular polymers, which use sequence and module character to establish elaborate behaviours [82].

Controlled polymerization also provides uniformity, which is important in studying and fine-tuning the structure–property relationships. If polymers have a narrow dispersity and predictable lengths of segments, it becomes possible to ascribe variation in material behaviour to changes in composition or architecture and not to a random distribution effect [83]. This transparency hastens the creation of materials whose thermal, mechanical, and chemical characteristics can be designed systematically. In addition, it is possible to combine polymers with controlled polymerization, which allows effective extension of chains and multiblock systems in which each block can be customized to add a particular function (e.g., environmental responsiveness, mechanical reinforcement, or segmental microphase separation) [84].

The other benefit of controlled polymerization is that it can be used with functional monomers with sensitive or stimuli-sensitive groups. Radical reactive intermediates tend to destroy or inactivate such functionalities by traditional radical polymerization because the radical intermediates are highly reactive [85]. By contrast, this is not the case with controlled radical mechanisms, wherein sensitive functionalities can be maintained, and this makes them part of the polymer backbone. This will be of special use in designing responsive polymers with a modular structure that achieves a very specific connection with the pH-sensitive, redox-active, or photoreactive motifs [86]. Similarly, regulated anionic, cationic, and ring-opening polymerization technologies offer a diversity in architecture because they allow the generation of uniform polyesters, polypeptides, and polysiloxane chains with excellent chain length and precision in their design [87].

Controlled polymerization is more than just a way of obtaining homogeneous polymers; it is a way to combine structural complexity and functionality, reliability, and precision. With a well-chosen choice of initiators, catalysts, chain transfer agents, and reaction conditions, chemists are able to programme polymer architectures that correspond to desired molecular designs of modular systems [88]. Controlled polymerization is the workhorse of polymer science, as multiple functional responsiveness- and application-directed behaviours become central as the defining feature of polymer science [89].

### 3.2. Post-Polymerization Modification Strategies

Although controlled polymerization forms the backbone of architected polymers, post-polymerization modification (PPM) adds an important new dimension of control, since, with this technique, chemists can precisely influence the functionality of polymers once the backbone polymer has been synthesized [90]. PPM systems enhance the flexibility of modular polymer systems by enabling them to selectively incorporate functional groups, responsive motifs, recognition units, or catalytic sites in sites that are hard to reach under polymerization alone [91]. This two-step synthesis, which allows the generation of a pre-defined backbone and then the subsequent functionalization of the same, offers a unique degree of customization that improves the versatility and multifunctionality of the resulting material [92].

The PPM methods are based on chemoselective reactions, which occur with high efficiency using mild conditions, allowing the preservation of the polymer backbone and saving the ability to modify it selectively [91]. The reactions that are used in this regard are the click chemistry reaction, thiol-ene ligation reaction, azide–alkyne cycloaddition reaction, Diels–Alder reaction, and nucleophilic substitution reaction. They generate high yields, low side products, and are resistant to extreme environmental conditions, and hence are the best to use in the modification of polymers that contain sensitive units or stimuli-responsive segments [93]. Notably, owing to the site-specific properties of PPM, functional groups could be presented with a controlled density and distribution, which is fundamental to obtaining the accurate macromolecular behaviour in modular systems [64]. The fact that PPM allows the decoupling of backbone synthesis and functional incorporation is one of the distinguishing advantages of the system. Such a separation enables investigators to obtain a generic, well-controlled structure of a polymer, which can subsequently be diversified into a variety of functional derivatives by simply changing the modification step [79]. This method is faster in the discovery of materials, as it allows one to systematically vary functional groups with a fixed architectural template. In the case of modular polymer design, it is especially beneficial, since different functional modules can be added one after another or orthogonally to form multi-module systems that independently but cooperatively carry out their functions [94].

The adaptability of the fine-tuning provided by PPM is essential in the formation of adaptive polymer systems that have dynamic reactions to external stimuli. To illustrate: By simply incorporating pendant groups that can be reversibly chemically modified by changes in pH, temperature, or redox conditions, it is possible to convert a hitherto passive polymer into an active, environmentally responsive material [95]. Equally, self-assembly behaviour, solubility, or interfacial interactions can be regulated by grafting hydrophobic, hydrophilic, or ionic units into specific locations. PPM has been applied in the biomedical field, allowing the incorporation of targeting ligands, therapeutic molecules, or biodegradation triggers without affecting the structural homogenization of the underlying polymer [96]. In sensor and coating applications, PPM has been used to introduce crosslinkable or photoreactive groups that offer access to the development of adaptive materials that respond under mechanical or chemical stimulus [97].

The other critical input that PPM has made on the modular design is the capability of providing multifunctionality in a hierarchical or spatially resolved way. In the case of functional groups located in particular regions, either on the backbone or as a cluster of side chains, the polymer may have complex behaviours depending on the localisation of those functionalities [98]. This improves control of module formation, chain folding, or microphase separation, all of which are needed in designing architected polymers with more advanced functionality like drug encapsulation, selective permeability, or catalytic activity [99]. More crucially, PPM has facilitated modification at the late stage, which entails that the same polymer scaffold can be and is adapted in various environments or end uses by merely grafting additional functionality and thus enhancing the versatility of materials and lowering the cost of synthesis [100].

PPM is thus an important design instrument in the manufacture of modular polymers, whereby structural accuracy, capability to adapt, and multifunctional combination are called upon concurrently [101]. PPM increases the design space of the next-generation responsive and application-oriented polymer systems by broadening the palette of possible functions beyond the functions available when only polymerization is involved [90].

### 3.3. Polymer Production Scalability, Efficiency, and Sustainability

With the growing role of modular and architected polymer systems in biomedical, environmental, and technological applications, the ease of scale, efficiency, and sustainability of synthetic processes are of growing importance [2]. Although laboratory-scale methods have their factors of value in proving the conceptual innovations, they have to be scaled up to the industrial scale without altering the structural accuracy as well as the functional integrity [102]. This translation must be performed using synthetic processes that are less harmful to the environment, are resource-efficient or optimized, and have strong control of the polymer architecture on a large scale. The modern-day green chemistry, catalytic efficiency, solvent selection, and continuous processing technologies are currently defining the future of strategies used to make polymers [103].

The transition to environmentally benign catalysts and reaction media can be considered as one of the most significant ones in the process of creating sustainable polymers. The conventional polymerization methods tend to standardize the use of toxic solvents, metal catalysts, or high-energy standards, which are natural environmental and safety hazards [104]. Conversely, the current synthetic strategies focus on minimizing or removing the use of hazardous reagents by using alternative solvents like water, bio-derived solvents, ionic liquids, or supercritical fluids. Such reaction environments not only provide ecological benefits, but also unique kinetic and thermodynamic benefits, which enhance uniformity of polymers, as well as suppress side reactions [105]. Otherwise, the catalyst systems are being re-engineered to run at reduced loadings, in less severe environments, or with recyclable and less toxic metals.

Scalability is also conditional upon the implementation of continuous and flow processes of polymerization, which are superior to conventional batch systems in terms of temperature, mixing, and reaction kinetics control [106]. Flow reactors permit polymerization under very controlled conditions that reduce batch-to-batch variation and permit reaction parameters to be adjusted very quickly. This level of control over the process is essential to reproducibility on a large scale in modular polymers that depend on high sequence control or highly sensitive functional groups [107]. Flow-based approaches also enhance safety, minimize the amount of waste produced, and allow on-demand synthesis, which in turn increases the overall sustainability of polymer manufacturing.

The other aspect of sustainable synthesis is energy reduction. The traditional controlled polymerization methods demand a lot of temperature regulation or prolonged reaction duration and drive up energy use and maintenance costs [108]. Present-day developments are aimed at decreasing the time of reaction by increasing the activity of a catalyst, using ambient-temperature polymerizations, and exploiting alternative energy sources, including light-driven or microwave-assisted reactions [109]. The photocontrolled polymerization has attracted interest more so because of its timeliness and lower heating demands. These processes are very compatible with the objectives of modular polymer production since sensitive functionalities are not lost, and side reactions are minimal due to such energy-efficient processes [110]. Also, on the environmental side, sustainability incorporates end-of-life behaviour, recyclability, and the idea of cycling. Contemporary synthesis of polymers is oriented more towards the use of the ability to reprocess, depolymerize, or biodegrade those materials under controlled conditions [111]. In the case of modular polymers, this entails the addition of linkages that are degradable and reversibly covalent, or monomers that can be recycled chemically into the architecture. These attributes are what guarantee the alignment of the lifecycle of the material with the concepts of the circular economy and environmental responsibility, without affecting the structural complexity and functionality needed in highly advanced applications [112].

**Figure 3:**
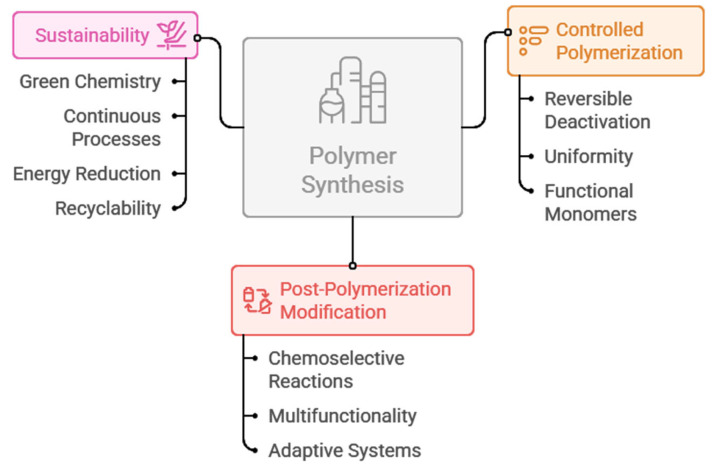
Advances in polymer synthesis: techniques and sustainability.

The inclusion of sustainability in the production of the modular polymers also improves cost-effectiveness, which should be part of mass adoption [113]. Efficient use of the monomers, high rate of conversion, reusability of the catalysts, and reduced purification processes all help in reducing the cost of production. In combination with the accuracy of controlled polymerization and the possibility of post-polymerization modification, sustainable manufacturing makes it possible to produce high-performance modular polymers that are economically and environmentally feasible [114] (Figure 3).

## 4. Stimuli-Responsive Polymer Systems

### 4.1. Fundamentals of Stimuli Responsiveness

Stimuli-responsive polymers are an advanced group of functional materials that are able to change their physical or chemical properties when a change is imposed on the environment [16]. Such changes might be changes in solubility, conformation, optical properties, mechanical behaviour, or chemical reactivity. Unlike the polymers of the past, where the properties of the polymers are largely fixed after the production process, responsive polymers mimic biological macromolecules in that they can sense, interpret, and respond to changes in the external environment [45]. This behaviour directly reflects the general aim of modular polymer design in which structural components are not only designed to fulfil a role partially in a static mode but also to be involved in active processes that demand the control of space and time [115].

The stimulus responsiveness is governed by the reversible interactions between the particular chemical groups and environmental stimuli. pH-responsive polymers, for example, are based on pendant acidic or basic functional groups that can be ionized or deionized based on the local acidity [116]. Electrostatic interactions and hydrophilicity are altered through ionization and affect polymer swelling, solubility, and conformation. The use of temperature-responsive polymers commonly takes advantage of the equilibrium between hydrophobic and hydrophilic interactions, e.g., in polymers that exhibit lower- or upper-critical solution temperature behaviour [117]. The polymer can go between collapsed and solvated states at temperatures either higher or lower than a critical point, permitting thermally controlled assembly, disassembly, or phase transitions.

Light-responsive polymers are polymers with chromophoric or photoactive groups, which can be addressed in a reversible way by being irradiated with a given wavelength [118]. These switches can undergo cistrans isomerization, bond fission, and crosslinking under UV light, allowing a highly non-invasive non-polymer behaviour to be controlled [119]. Conversely, redox-responsive materials are polymers with motifs that are sensitive to oxidative or reducing conditions, such that the polymer can sense biochemical or cellular gradients or chemically reducing agents. The combination of these various stimuli provides an expansive approach to design opportunities in which polymers may be specialized to work under extremely specific conditions [120].

The key attribute of stimuli-responsive polymers is that the response is reversible, meaning that the material can be subjected to repetitive cycles of activation without damage [32]. This dynamic equilibrium is required in applications where the polymers need to be able to cycle between responsive states many times, e.g., in drug delivery systems that need to release therapeutic agents on demand, or self-regulating materials that need to be able to restore functionality after damage. Since environmental signals can be highly variable, the capability of the polymer to change between states without discontinuity will dictate the reliability and efficiency of the polymer in practice [121]. The molecular structure and spatial arrangement of responsive units are also important in responsive behaviour. This is greatly improved by the modular design framework, as it allows stimuli-sensitive groups to be localized in the polymer network, backbone, or side chains. This encourages overall consistent and collaborative reactions throughout the content [55]. Modular placement also affects the reaction rate of the polymer to stimuli and the magnitude of response, as well as the stability of the intermediate states. Consequently, stimuli-responsive polymer bridges a fundamental crossover between molecular engineering, materials science, and application-focused innovation, which makes them essential building blocks of new-generation polymer systems [122].

### 4.2. Architectural Design Strategies of Responsive Architectures

To achieve the design of polymer structures with controlled responsiveness, a systematic combination of chemical motifs capable of selective interaction with environmental stimuli is necessary. In modular polymer systems, the process of integration is performed by dispersing responsive elements in a designated range of the polymer structure [123]. Any module is selected due to its intrinsic sensitivity to a particular stimulus and the fact that it can be compatible with other segments in the polymer. The main issue is to create a balance between the level of responsiveness, structural stability, and reversibility of the transitions in such a way that the material can operate in complex environments in predictable manners [124].

The first basic approach is to choose responsive motifs, which can be experimentally transformed by reversible chemical or physical reactions whilst preserving integrity in ambient conditions. The addition of ionizable groups, e.g., carboxylates or amines, enables the polymer to adjust to pH changes, whereas the integration of thermosensitive units like ethylene oxide or hydrophobic blocks enables one to have controlled transitions with respect to temperature [125]. Equally, photo-sensitivity may be added by the addition of azobenzene, spiropyran, or coumarin analogues, and reversible redox switching by the addition of redox-active groups, including disulfides or ferrocene-based groups. These units are incorporated in the modular structure by a controlled polymerization or post-polymerization modification to ensure that the fit of the structural units together adds greater overall capability to the material [126]. Another design aspect that is critically considered is the density and distribution of the responsive motifs. Sparse distribution can result in incomplete transitions or heterogeneous behaviour, as well as incomplete transitions being described by high densities, which may also shorten response times and destabilize structures [127]. A way out is in the modular design, which allows these motifs to be located in non-overlapping yet interacting areas. Placed at block junctions, or crosslinking junctions, or side chains, responsive units can enhance the influence of environmental changes, producing a more pronounced transition without sacrificing the strength of the material [128].

A trade-off between responsiveness and stability is especially critical in applications in which polymers should be able to endure any physical or chemical pressure and remain flexible [129]. This involves stabilizing components that balance the dynamic behaviour of the responsive modules. As an example, a thermo-responsive polymer can be designed to retain mechanical integrity even on repeated transitions by adding rigid aromatic units, and to stabilize pH-responsive conformational changes by the addition of a hydrophilic unit [36]. This balance depends on the fact that the module interactions should be comprehended, and the overall architecture should be taken into consideration. Adaptable positioning will make sure that responsive and stabilizing units complement each other instead of competing with each other [130].

The second important factor that characterizes a well-designed responsive architecture is reversibility. In most applications, e.g., targeted drug release or adaptive coatings, reversible transitions would have a polymer revert to its starting position once the stimulus is taken away [131]. To create reversible systems, it is necessary to design motifs with predictable signal response interactions and not to have irreversible crosslinking or degradation events. Reversible responsiveness in a modular design may be improved through dynamic covalent chemistry or supramolecular interactions, which may be rearranged under mild conditions [132]. Controlled polymerization and computational modelling can also be used to provide predictability to the design of responsive architectures. In cases where the molecular geometry is known, the effect of alterations in conformation, energy landscapes, and responses to environmental changes can be predicted by using computational tools to simulate changes in conformation, energy landscapes, and environmental effects [133]. This predictive power speeds up the process of identifying the best motifs and structures to be used in certain applications, and the end product of such a process is a material that would behave trustworthily in the desired conditions. Due to the combination of computational knowledge with modular synthetic methods, researchers can produce functional, responsive polymer systems that are structurally well-developed and functionally advanced (Table 2) [134].

### 4.3. Synergistic Responsiveness in Multifunctional Polymers

Although single-stimulus-responsive polymers are useful in their own right, having many responsive units in one molecular structure greatly increases the adaptive abilities of the material. Multifunctional polymers formed by mixtures of pH-, temperature-, light-, and redox-responsive units can perform complex behaviours that can be compared to natural adaptive systems [135]. These modular architectures are not merely individual responses but produce synergistic behaviours where one module impacts or heightens the response of another module. This collaborative process leads to material that is multi-stage transitioning, that is, with sequential responding, or cross-regulated behaviour based on the environmental interface [136].

Synergistic responsiveness comes about when responsive motives are arranged in a manner that the transitions are made to interact in a positive manner. As an example, a polymer carrying a thermo-responsive and pH-responsive segment can only display higher swelling at higher temperatures when maintained at a given pH, which forms a dual-gated process that can be only activated under strictly controlled conditions [137]. Likewise, by incorporating light-responsive entities close to redox-reactive units, photochemical switching can be used to control electron transfer, and complex optical and electrochemical behaviour can be imposed. All these synergistic interactions allow the polymer to work at some highly sophisticated functions like controlled activation, multi-step release, or selective permeability [138]. Such multi-module systems should be designed in consideration of how responsive units can be made to interact across molecular scales. The responsive motifs can be inserted in segregated areas in the modular architectures, which transition independently or cooperatively. The transitions can spread through the material when modules are purposely set up to couple with one another, e.g., by the positioning of thermosensitive blocks near light-sensitive groups, producing large-scale structural changes at the basis of localized stimuli [139]. The given phenomenon is especially applicable in designing actuators, environmental sensors, and materials that can be morphed.

The other attribute of the synergistic responsiveness is the increase in multifunctionality. A polymer with more than one responsive unit can serve a number of functions at the same time and act as a sensor, carrier, stabilizer, or catalyst according to the environmental situation [23]. As an example, biomedically oriented polymers can be prepared by making use of pH-sensitive units that deliver therapeutic agents in acidic conditions and using temperature- or redox-responsive units to control secondary behaviours such as degradation or intracellular penetration. Multifunctional polymers in materials engineering [140]. Multifunctional polymers can change their mechanical behaviour or surface properties in response to a combination of stimuli, allowing self-healing coatings, controllable wettability, or sensitization to foulage.

**Figure 4:**
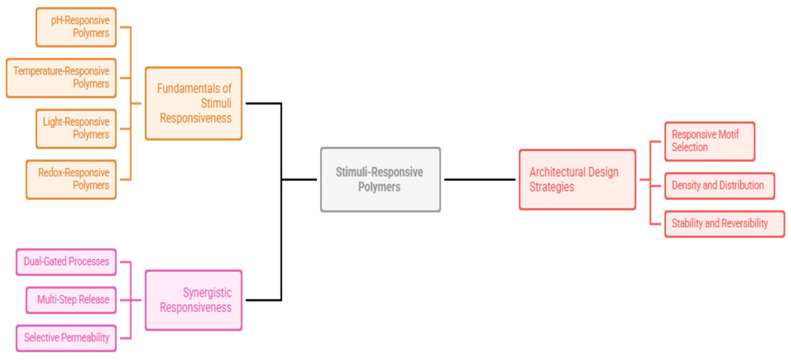
Stimuli-responsive polymer systems highlight the relationship between the basic stimulus (pH, temperature, light, and redox) and their architectural design approaches, as well as the resultant functional behaviours. The figure demonstrates that responsive motif choice, density, distribution, and stability enable a synergistic response, including a dual-gated process, multi-step release, and selective permeability, through a modular polymer design.

Multifunctional polymers are also characterized by multiple signals in a coherent design, which improves selectivity and precision [141]. They do not react to one environmental feature, but a combination of conditions to trigger, and false triggers are minimized, and reliability is enhanced. This is essential in complicated systems like the human body, where several biochemical parameters vary concurrently, or in environmental systems where chemical gradients, as well as physical stimuli, interact [142] (Table 3) (Figure 4).

## 5. Application-Oriented Innovation by Modular and Responsive Polymers

### 5.1. Specific Drug Delivery Systems

The development of modular and stimuli-responsive polymers has become one of the most useful technologies in targeted drug delivery, due to their capacity to combine the specificity of structural organization with the responsiveness of behaviour [147]. These systems can specifically respond to the complex biochemical environments found in vivo because the ability to encode environmental sensing units directly into the polymer structure enables these systems to intelligently interact with their environment. In controlled delivery platforms, the polymer has to be stable when circulating, protect its cargo against premature degradation, and disperse the therapeutic cargo under the conditions that are required [148]. Modular design can give the structural control needed to fine-tune each of these functions, and stimuli responsiveness can make release selective to physiological signals (pH gradients, enzymatic activity, redox potential, or temperature changes).

The effectiveness of environment-driven release is based on the location of responsive modules, which undergo reversible transition, e.g., pH-sensitive units may be embedded in the polymer mesh such that release of drugs is induced when exposed to the acid microenvironments that occur in inflamed tissues or tumour locations [149]. On the same note, redox-independent linkages are very suitable in intracellular drug delivery, where high concentrations of glutathione can trigger bond rupture and release in the cytoplasm. Hypothermic therapy hyperthermia-assisted therapies can utilize the property of thermal responsiveness: thermally induced heating causes the polymer to contract or expand, and so can be used to control the diffusion of drugs [150].

Modular architectures are an extra degree of accuracy because they allow the creation of sufficiently defined micelles, vesicles, hydrogels, and nanocarriers of controllable size, morphology, and cargo-loading capability [151]. Such a level of control is necessary to attain the best biodistribution and to reduce off-target effects. The choice of blocks to create self-assembled nanostructures allows the researchers to design a nanostructure that traps therapeutics effectively and is stable in biological fluids by choosing hydrophilic or hydrophobic blocks. The targeting ligands (peptides, antibodies, or small-molecule recognition units) can also be incorporated during the spatial organization of functional segments, which increases specificity to diseased tissues. Modularity and responsiveness therefore contribute to the development of drug carriers with precisely controlled release kinetics, high biological permissibility, and contextual activation [152]. Such attributes are important in the current therapeutic approaches, such as cancer therapy, targeted protein delivery, gene regulation systems, and regenerative medicine, in which the ability to achieve specific spatiotemporal control of bioactive agents contributes greatly to the outcome of therapeutic intervention [153].

### 5.2. Self-Healing Polymer Systems

Self-healing polymers are a category of materials that are developed to self-repair structural damage and recover their functionality. They have been applied to biomedical implants, structural elements, and protective surfaces [154]. Combining the principles of modular design with responsive functionality is a very significant boost in the ability of self-healing polymers to respond fast and efficiently to mechanical or chemical insults. Older self-healing mechanisms tend to be based on encapsulated agents of healing or irreversible chemical processes, which reduce lifetime. Unlike this, modular-responsive systems directly integrate reversible interactions or dynamic covalent bonds into the polymer backbone such that repeated cycles of healing can occur, without exhausting the reactive materials [62].

Modularity of the architecture gives the ability to distribute healing motifs within the polymer network instead of localizing them in a few isolated microcapsules. This random distribution has the effect that any damage that takes place, e.g., in terms of cracking, abrasion, or stress-induced separation, can be repaired by the responsive modules reorganizing, reforming bonds, or initiating local transitions, which are used to repair the damage [155]. As an illustration, self-healing, hydrogen bonding-based self-healing, metal–ligand coordination, π–π interactions, and reversible covalent reactions can be included in the modular framework in a strategic way to provide mechanical strength and flexibility [156].

The stimuli are essential in the activation of the healing process. When appropriate temperature-responsive segments are used, the latter can soften or flow when heated, and, as a result, the polymer chains can re-entangle and regain mechanical integrity [157]. Photoreactive units are light-responsive and can react to controlled reactions triggered by light and cause re-crosslinking in the damaged regions. Redox-responsive groups can rearrange or restructure network connectivity in oxidative conditions [158]. These units are then used together with reinforcing structural blocks, which give the resulting materials both strength and dynamic healing properties.

The benefit behind the inclusion of modular motifs is the fact that it is possible to independently tune the healing effectiveness, activation threshold, and mechanical characteristics [159]. As an example, the speed of healing can be increased by increasing the concentration of dynamic bonds, but weakness might arise, or better durability can be achieved by adding rigid segments without losing responsiveness of the motifs to reorganize under relevant stimuli [160]. It is through this interaction that researchers can come up with self-healing systems that can be optimized to suit specific applications, be it fast healing at ambient temperatures, high mechanical strength, or long life under stress. Using this synergy, modular and responsive self-healing polymers have enormous potential in enhancing the life of materials, lowering maintenance needs, and making them more sustainable in an incredibly diverse range of technological and biomedical studies [161].

### 5.3. Adaptive and Smart Coatings

The creation of adaptive coatings is one of the most exciting fields of application of modular and stimuli-responsive polymers. These coatings are designed to detect and react to their surrounding environment and change their surface properties to increase protection, performance, or functionality [32]. Their dynamic properties enable surfaces to change dynamically in response to changes in pH, temperature, humidity, mechanical stress, or chemical exposure, and such adaptability is much better than that of conventional unresponsive coatings [162].

In modular-responsive coating, the architecture is shaped to incorporate responsiveness in the polymer backbone. Responsiveness groups such as pH can be incorporated to respond to the acidity in the environment, changing both the surface charge and hydrophilicity, which is especially useful in corrosion prevention and anti-fouling [116,163]. Coating elasticity or permeability can be regulated in a thermal-responsive segment to be able to react to temperature variations, allowing, e.g., thermal control or self-cleaning. This is made possible by light-responsive units that can be incorporated near the surface to enable careful adjustments of wettability, crosslink density, or colour when exposed to certain wavelengths [164]. The modular design allows these functional groups to be located strategically to ensure the stimuli-responsive behaviour is increased at the point of interaction with the environment [128]. The polymer network underneath may include reinforcing modules that are mechanically stable, such that the active surface is not destroyed as the process of expansion, contraction, or chemical interaction is repeated. This structural–functional hierarchy is reminiscent of natural systems like those found on the skin or plant surface, where there is a modular layering that gives the system some protection and some form of adaptability as well [165].

Smart coatings have other advantages besides mechanical or chemical protection. The responsive coating can control adhesion, biofouling, and optical reflectivity, or can release protective agents in a controlled fashion [166]. An example is a marine coating that can act on biological adhesion by changing the hydrophilicity of the surface or exposing anti-fouling compounds to chemical signals of microorganisms. Coatings in industrial applications can be used to alter the permeability or hardness of the surface to prevent corrosion or abrasion of the underlying material. Since the modular architectures have high structural integrity, such coatings can bear repeated cycles of stimulus-induced adaptation without degenerating [167].

### 5.4. Polymer-Based Sensors

Polymer sensors take advantage of the fact that stimuli-responsive materials have the capacity to convert changes in the environment into a measurable signal. Modular-responsive architectures provide special benefits to sensing applications, due to a combination of finely tunable structure with selective responsiveness [168]. Incorporating the responsive motifs into an established polymeric system offers a flexible system on which chemical, biological, mechanical, and optical sensors can be developed [55].

Changes caused by stimuli in such systems could be in optical properties, changes in electrical conductivity, changes in mechanical behaviour, or swelling and permeability changes. These changes are facilitated by the modularity of the polymer to be amplified or pointed towards a certain mode of detection [25]. As an illustration, the colourimetric or fluorescence change can be very specific by incorporating chromophoric units in areas that experience a conformational change in relation to the analytes. Adding blocks with conductivity to the surroundings of ionizable units allows the production of sensors with an electrical response dependent on the pH or ionic strength [169].

The cooperative behaviour of modular functional units is what causes the sensitivity of these polymeric sensors. In the case of responsive motifs dispersed in the polymer matrix, collective transitions are caused by small environmental variations and enhance the signal [57]. This magnification is very important in the detection of low concentrations of analytes or weak physical stimuli. In addition, the modularity will provide the ability to evolve responsiveness to particular targets through the adjustment of the identity or dispersion of recognition units [170]. Functional groups that selectively complex metal ions, biomarkers, pollutants, or metabolites can be added to polymers, which allows sensing of a wide range of targets in a highly selective manner.

**Figure 5:**
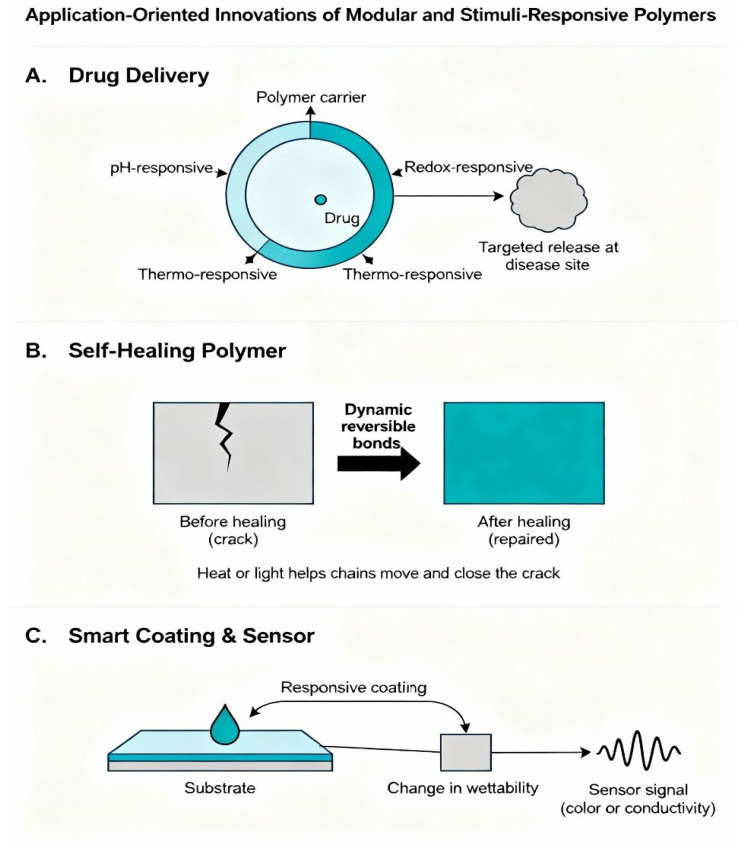
Schematic illustration of modular and stimuli-responsive polymer applications: (**A**) targeted drug delivery with responsive release, (**B**) self-healing polymer systems, and (**C**) smart coatings and sensors for adaptive responses.

Wearable devices, flexible electronics, environmental monitoring, and biomedical diagnostics are also good applications of integrated sensing systems that are constructed using modular polymers [171]. Their capacity to balance both adaptability and mechanical flexibility and biocompatibility is what differentiates them compared to inorganic sensors, which in most cases do not have the conformational freedom needed to adapt to dynamic conditions [172] (Figure 5).

### 5.5. Cross-Sector Applications: Biomedicine, Energy, and Environment

Their flexibility allows one to reuse and repurpose modular and stimuli-responsive polymers in a wide variety of modules in biomedicine, sustainable energy systems, and environmental technologies. They are universal in that they can be engineered to form molecular structures that are structurally precise and that interact dynamically with complex environments [16,55].

In biomedicine, the polymers aid in the deployment of therapeutic vehicles as well as implantable devices, in addition to tissue scaffolds and diagnostic platforms. They can react intelligently to biological systems due to their biocompatibility, their ability to control degradation, and their ability to behave context-dependently [173]. Modular architectures can incorporate cell-instructive motifs, antimicrobial units, or mechanical reinforcements, whereas responsiveness offers adaptive behaviour, required to support real-time therapeutic or diagnostic operations [174]. Stimuli-responsive polymer is also used in the development of advanced energy storage materials, adaptive membranes to be used in separating processes, and electrolytes in sustainable energy technologies [175]. Their modularity allows them to dictate ionic conductivity, mechanical strength, and thermal stability with high precision, whereas stimuli responsiveness adds the functions of self-regulation of an ion flow, adaptive permeability, or photoinduced changes in conductivity. These are essential in the next generation of batteries, fuel cells, and photovoltaic systems [176].

**Figure 6:**
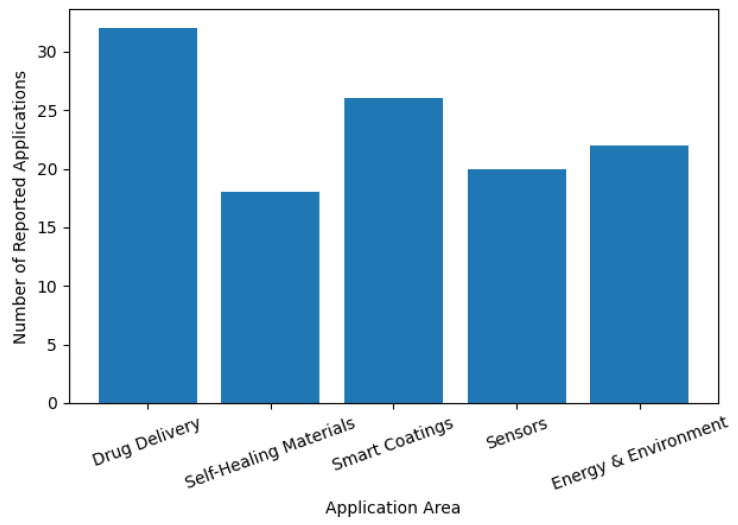
Histogram illustrating the application-wise distribution of modular and stimuli-responsive polymer systems reported in the literature. Drug delivery and adaptive coatings emerge as dominant application domains, while growing interest is observed in sensing and energy–environmental technologies.

Responsive polymers can capture, release, or degrade pollutants in response to environmental cues, allowing controlled remediation methods [177]. The selective binding sites and degradable or recyclable modules can be incorporated in modular structures, which is in line with sustainability [178]. Adaptive membranes can be used in the cleaning of water, where the pore size can change when the solutes change to increase the filtration efficiency. Responsive coatings can be used in air treatment systems to eliminate acidic or oxidative pollutants by means of stimulus-induced reactions [179]. In all these aspects, the theme of universality and environmentally friendly design is kept in focus. Not only are high-functionality polymers enabled, but also stimuli-responsive and modular polymers have routes to sustainable manufacture, controlled degradation, and circular use [180]. Their versatility in working through biological, chemical, and physical interfaces makes them the basis of the future generation of adaptive technologies (Table 4). In order to give a quantitative picture of the modular and stimuli-responsive polymers application landscape, a histogram was used to sum up the distribution of reported applications in the various sectors, and these are presented in Figure 6.

## 6. Computational and Machine Learning Approaches for Polymer Design

### 6.1. Computational Modelling of Rational Design

Computational modelling has emerged as an essential part of contemporary polymer science, delivering both mechanistic information and prediction ability that do not negate experimental synthesis [185]. With the increasing complexity of polymer structures, particularly in modular and stimuli-responsive structures, the use of computational methods provides a methodical way of exploring polymer structural possibilities, predicting functional behaviour, and finding the best design parameters in vivo before laboratory synthesis [186]. Simulations have been useful in explaining how molecular arrangement, location of functional groups, and segmental dynamics help in the rational design of architected polymers and their emergent material properties [187].

Coupled with molecular dynamics, coarse-grained simulations, quantum chemical calculations, and density functional theory, a multiscale insight into the behaviour of the polymer is obtained. On a molecular level, simulations are able to predict chain conformation, bond rotation, and intramolecular interactions, which are needed in designing responsive modules that experience controlled transitions [188]. Computational tools at the mesoscale map the arrangement of polymer chains into micelles, hydrogels, networks, or phase-separated architecture, providing understanding of the stability of the structure, its permeability, and responsiveness to different environmental conditions. Hierarchical modelling is especially beneficial in modular systems, where several functional segments engage in a synergetic interaction, and the final arrangement of such a system determines the ultimate material properties [189]. Amongst the most significant benefits of computational design is the fact that it allows one to ask questions regarding how particular changes that are caused by a specific stimulus spread throughout a polymer structure [190]. Indicatively, the process of protonation of a pH-reactive unit can be predicted through modelling to understand the changes in local hydrophilicity (as a result of protonation) and consequently in global swelling. On the same note, light-responsive motifs should be simulated to understand the paths of energy transfer, the efficacy of photochemical conversion, or the restructuring of the structure upon light illumination. The knowledge allows the researchers to anticipate the level of responsiveness, adjust the activation energies, and regulate the extent and reversibility of the transition of the material [126].

The sustainability aspects that characterize the next-generation polymer design are also best assessed using computational modelling. By simulating synthetic sequences with reduced energy requirements, the degradation behaviour, and the ability to simulate the development of modular architectures in a manner that is either recyclable or can be depolymerized, can be identified [191]. With the incorporation of chemical reactivity calculations and thermodynamic stability calculations, computational algorithms enable scientists to construct polymers that fulfil functional, structural, and environmental goals [192].

Finally, rational computational modelling gives a roadmap through which synthetic strategies, the location of responsive modules, and the predictability of material performance are informed [193]. The combination of theory and experimentation with this synergy increases the development of highly functional polymer systems, as well as lessening the requirement of conducting many trial-and-error experiments in synthesis [194].

### 6.2. Machine Learning for Structure–Property Prediction

Machine learning (ML) has also significantly increased the range of computational polymer research by allowing predictions of structure–property relationships using data in large chemical design spaces [195]. Since countless combinations of monomers, functional modules, and architectural patterns can be investigated with modular polymers, ML is an especially effective method of finding the most optimal designs that can be used to achieve particular performance specifications. Conventional computational techniques use a direct physical model, but the ML algorithms are trained on datasets, which enables them to learn complex relationships that are potentially hard to determine from first principles [196].

ML models can be trained to predict the properties of polymers, including glass transition temperatures, mechanical strength, degradation behaviour, solubility, responsiveness limits, and self-assembly behaviours, by training on experimental or computational data [197]. ML is particularly good at determining the effect that small changes in the identity or the distribution of functional groups have on responsiveness in the context of modular and stimuli-responsive polymers. An illustration is that algorithms can determine the best ratio of hydrophilic and hydrophobic groups to be temperature-sensitive, or specific sequences of monomers that lead to swelling on demand or catalyze the breaking of bonds during redox reactions [198].

The feature engineering is important in the representation of the chemical and structural descriptors, which control polymer behaviour. Even complex networks spanning multiple functional modules can be understood by their representations as molecular fingerprints, graph embeddings, or vectors of descriptors, which can be used in ML models [199]. Graph neural networks, transformer-based architectures, and other deep learning models have demonstrated impressive success in predicting polymer properties with high quality, as they can model polymers as structured networks instead of simple linear formulas [200]. In addition to predicting properties, ML can be used in inverse design, in which specifications regarding the desired functional properties are provided, and the algorithm is used to produce the possible polymer architecture that would meet those specifications [201]. This has been revolutionary in the design of modular polymers, since researchers are now able to experiment with unusual combinations of monomers, or even unusual architectural motifs that could not necessarily have been tested as an experiment. Variational autoencoders and multifunctional generative adversarial networks are examples of ML-driven generative models that can offer new macromolecular structures with desired responsiveness and multifunctionality [202]. Besides the ability to design, the ML improves accuracy by detecting synthetic bottlenecks, or telling whether a particular set of monomers can be used in a certain polymerization technique. These predictive understandings facilitate the planning of the experiment process and decrease the probability of synthetic failure [203]. As data accessibility keeps growing, ML tools will be able to be used in designing advanced polymer systems incorporating the complexity of structure and the precision of responsiveness.

#### Advantages of Machine Learning in Polymer Design

Machine learning (ML) can quickly extract missing structure–property relationships in large and complex polymer datasets, in contrast to traditional trial-and-error methods or completely physics-based simulations. Polymer systems that are architected can have high-dimensional design spaces, such as monomer sequence, block length, topology, functional group density, and processing conditions. ML models are especially well-suited in this kind of system, since they can model nonlinear interactions that cannot be resolved with analytical or empirical models alone [204].

One of the advantages of ML is that it forecasts material performance before its synthesis, and this saves on experimental cost, time, and material. The trained ML models have the potential to screen thousands of possible polymer architectures in a short period of time and isolate promising candidates with specific mechanical, thermal, electrical, or responsive behaviours. This predictive ability is particularly useful in the case of modular polymer systems, in which functional differences can be large due to small changes in the architecture [205].

There is also the ability of machine learning to design the desired macroscopic properties, where the desired properties are defined initially, and then the model proposes the preferred polymer structures. This methodology takes the polymer development away from descriptive knowledge and towards objective-focused engineering. Moreover, the ML frameworks can be constantly upgraded with the help of new experimental data as they appear; therefore, they are flexible tools of changing material platforms instead of fixed predictive models [206]. ML has been useful in the context of responsive and multifunctional polymers, particularly in bridging between molecular design and performance requirements in the real world, facilitating the transformation of complex design concepts into materials realistically accessible to experimentation (Table 5).

### 6.3. Discovery Rush and Sustainable Minimization

Data-driven polymer discovery is becoming an extremely rapid process with the integration of computational modelling and machine learning. In the past, creating a novel polymer used to take many phases of synthesis, characterization, and refinement [207]. With their enormous combinatorial possibilities, the modular architectures would be particularly hard to effectively optimize by experimentation only. Computational tools solve these problems by quickly screening candidate structures, making predictions of important properties, and eliminating vast design spaces before laboratory testing. This speed saves resource usage and greatly cuts the development timeframes [208].

Among other effects, computational approaches have provided one of the most effective directions in sustainable polymer design. Computational methods enable the development of materials in an environmentally friendly manner by helping to locate synthetic pathways with lower energy requirements, catalysts with lower energy needs, or less solvent waste [209]. ML models have the potential to forecast degradation mechanisms, risks of bioaccumulation, and recyclability characteristics, allowing polymers to be designed circularly from the beginning. Computational sustainability assessment is especially helpful in modular systems, where the building blocks can be recombined or redesigned [210]. It enables the comparison of alternative architectures in a preferred way, both quickly and quantitatively.

Adaptive material development is also supported by the accelerated optimization by means of computational methods [211]. As an illustration, the incorporation of stimuli-responsive motifs in a modular structure means that responsiveness, mechanical stability, and durability must be balanced. These multidimensional trade-offs can be explored by ML algorithms, and the designs that produce an optimal compromise can be found. The behaviour predicted can then be checked by simulation to check the behaviour under conditions that are simulated to imitate real application conditions [212]. By doing this, a more effective load is minimized in the experiment, and more confidence is gained in the performance of the final material.

**Figure 7:**
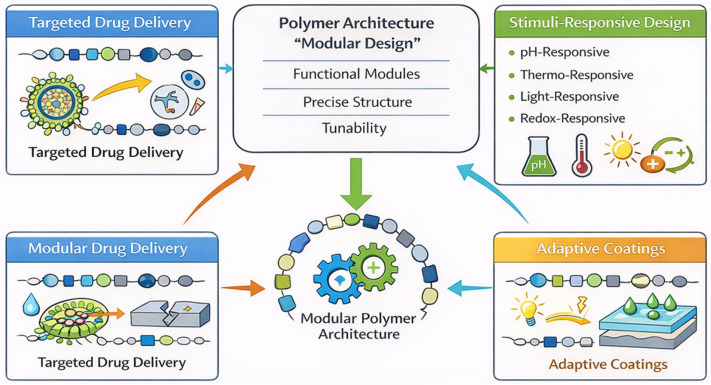
The diagram illustrates application-oriented polymer design, featuring a central modular polymer architecture with tunable functional modules linked to targeted/modular drug delivery, stimulus-responsive designs (thermo-, redox-, pH-, light-responsive), and adaptive coatings.

Also, computationally screened sets can be used to find underutilized chemistries and structural motifs, which may be more responsive, more multifunctional, or more sustainable [213]. The insights are used to introduce innovations because they increase the design space beyond the heuristics that have been the norm in polymer science. In conjunction with the accuracy of controlled polymerization and the ease of post-polymerization modification, calculation techniques make modular and responsive polymers a fast-developing category of materials with enormous technological applications [214] (Figure 7).

## 7. Integrative Perspective: Bridging Synthesis, Responsiveness, and Application

### 7.1. How Modular Synthesis Drives Multifunctional Responsiveness

Historical developments in modular polymer synthesis have essentially transformed the conceptualization of functional materials and have permitted the creation of a direct and rational connection between molecular architecture, stimuli responsiveness, and emergent functionality [215]. Modular architectures, in contrast to conventional polymer systems, in which the association between structure and behaviour is commonly obscured due to the compositional randomness, are characterized by the careful placement of functional modules as well as the controlled distribution of sequence [82]. This structural deliberateness guarantees that every part has a distinct purpose in the larger system, and this is the same type of organization that provides the next-generation polymers with their responsiveness and multifunctionality [216].

In modular structures, the architectural design predetermines the connection between the stimuli-sensitive units and the polymer structure and the distribution of the transformation through the material [217]. The response of the polymer to environmental stimuli can be predicted and programmed when responsive motifs are present in specific locations. An example is the sharp thermo-responsive transitions made by placing hydrophobic and hydrophilic segments in a defined sequence, and the sharp swelling or contraction of groups of pH-sensitive ones by clustering. The architecture, therefore, controls both the size and the dynamics of the response, making it possible to fine-tune the response in order to achieve application-specific performance [218].

This correlation between structure and responsiveness is yet another significant correlation in the design of multifunctional polymers. This is because, with modular structures, it is possible to have independent responsive units assembled into cooperative structures, resulting in synergistic behaviours that cannot be attained under random copolymerization [18]. These interactions allow materials to react to more than one stimulus hierarchically or sequentially, and generate adaptive responses that are complex, like biological systems. As one example, a polymer with redox-responsive linkages embedded in a temperature-responsive matrix can be rearranged structurally in only combined oxidative and thermal conditions, providing dual-level control, which is very desirable in targeted drug delivery or in adaptive coatings [219].

In addition, modular synthesis encourages a direct route between chemical structure and functionality related to the application. Since every module still maintains its character and provides a specific functionality, the resulting material properties can be linked to design parameters, which were set on the molecular scale [220]. The given clarity serves to speed up the optimization procedure and increase the reproducibility demanded by the practical application. It is the architectural design that is then one of the central means through which responsiveness is not only attained but also through which the responsiveness may be converted into meaningful technological operations [221].

### 7.2. Synergy Between Synthetic Precision and Application Demand

Combined with the controlled polymerization method and the principles of the modular design, the convergence of these two has formed a synthetic platform that is exceptionally well-suited to the swiftly growing needs of advanced technologies [222]. Controlled polymerization provides uniformity in chain length, sequences that can be predicted, and high structural fidelity, whereas modularity enables functional units to be integrated in a manner that is supportive of the operational needs of a variety of applications. This synergy allows the production of high-performance materials, the performance of which is directly correlated with the precision of their synthesis [19].

The growing and developing technologies require more and more materials that can work well in unstable environmental conditions, react dynamically to changes within the environment, and have several functions at the same time [16]. Responsive polymers with a modular design are in a better position to address these requirements. In medicine, materials should be biocompatible with selective reactivity and degrade in a controlled manner. These properties can be programmed by using controlled polymerization methods such as degradable blocks, hydrophilic segments, or bioactive motifs in controlled arrangements. In drug delivery systems, this accuracy takes care of the fact that the responsiveness to pH, temperature, or redox gradients is closely controlled, reducing the premature release and maximizing therapeutic efficacy [85]. The requirement of adaptive behaviour is also high in energy and environmental technologies. Polymer electrolytes have to react to ion concentration changes, membranes have to dynamically control the permeability, and protective coating has to vary its surface properties to avoid degradation [223]. These functions are facilitated by controlled polymerization, which allows ordering of the segments, controlled distance between modules, and control of the crosslink density. Such materials, when mixed with modular responsiveness, gain the ability to carry out complicated functions, including selective ion transport, self-repair, or adaptive shields [224].

Synthetic precision and application demand also provide the driving power of innovation since such materials can be tailored to niche or extreme conditions. As an example, for a polymer to be used in space exploration, in the ocean ecosystem, and in biomedical implantation, patients should be able to function in extreme or variable conditions [225]. The modular design is such that responsive parts are not killed during the processes, and structural modules are intact. Driven synthesis ensures these units are maintained in stable, predictable conformations so that the polymer can operate in a reliable regime even in challenging environments [55].

Additionally, this synergy can be further boosted by the incorporation of computational models and machine learning, which allow for the making of predictive information on structure–function relationships. Synthetic paths can be selected to fit the performance measures that are predicted, and modular components can be set up in the way that has been identified as optimum by the computation. This synthesis–precision matching prediction on theory accelerates the production of materials that satisfy intricate application-driven criteria [226].

### 7.3. Challenges and Design Principles for Next-Generation Smart Polymers

Modular polymers and stimuli-responsive polymers are yet to reach their full potential in next-generation technologies; however, there are certain challenges that should be overcome. These issues are connected through the combination of themes mentioned in the abstract, such as multifunctionality, sustainability, responsiveness, and predictive design, and each of them could be used to enhance and enlarge the existing methods [227]. The introduction of multifunctionality is very desirable; however, it creates complexity in the synthesis, characterization, and performance prediction. It is becoming harder to ensure that various responsive units are being incorporated into a single architecture work cooperatively and not competitively [48]. To surmount such a difficulty, the tenets of hierarchical design, which organize modules to be functionally independent yet integrated to give rise to integrated behaviour, are needed. This balance requires close control over block position, the distribution of the lengths of segments, and the potential of the interactions, which require development in controlled polymerization and post-polymerization modification.

The other important consideration is sustainability. Synthesis of architected polymers is typically an energy-intensive, special-purpose catalyst or non-renewable monomer. The upcoming design approaches should include a requirement to include the principles of green chemistry in their design, and the selection of monomeric units should be bio-derived, recyclable, or biodegradable [228]. This can be supported with the help of computational tools that predict more environmentally friendly synthetic paths and come up with recyclable architectures preserving structural flexibility. Sustainability design can also mean taking into account end-of-life behaviour, whether through controlled degradation, depolymerization, or mechanical recycling, and ensuring that modularity is also circular instead of circularizing it [112].

The next-generation polymers also need to be more responsive, not limited to on-and-off switching behaviours, but with more detailed, graded, or multi-stage behaviours. To reach this level of responsiveness, it is necessary to combine motifs to react to minute variations in the environment and retain the reversibility and stability of the structure [23]. The difficulty is to design such motifs in modular designs without reducing the mechanical strength or lifetime of service. Some of the strategies can involve the use of dynamic covalent chemistry, supramolecular interactions, or more complicated multifunctional systems that can cascade responses [229].

Predictive design is the foundation of future innovation. Conventional experimental methodologies are unable to explore the vast design space that is produced by the combination of modular synthesis and multifunctionality [230]. The process of design should then be incorporated with computational modelling and machine learning, which will allow for predicting the responsiveness, degradation profile, mechanical performance, and environmental compatibility. The difficulty is to produce high-quality datasets, create the right polymer representations, and close the gap with the simulation and experimental validation. However, the tools that can be used to predict this will be vital in speeding up the discovery of solutions as well as ensuring that next-generation polymers achieve both the performance and sustainability standards [231]. All these issues point to the necessity of integrating a comprehensive approach to polymer design—that is, one that combines molecular structure, environmental friendliness, functional versatility, and computing forecast [114]. Using these mutually reinforcing concepts, scientists will be able to create intelligent polymer systems that are not only multifunctional and responsive but also sustainable and specifically adapted to novel uses [15].

#### Quantitative Design Principles for Smart Polymer Architectures

To address these obstacles, various quantitative design principles have become the central guiding principles of the next-generation smart polymer systems. Responsive module density is one important parameter that will directly affect the magnitude and rate of transition to stimuli. Either underdensity or overdensity can lead to poor or incomplete response, and overdensity can cause destabilization of the polymer network. Designing this will be optimal when it involves finding density windows that are responsive and do not threaten the durability. Response threshold is also another crucial parameter, which is described as the lowest level of stimulus that causes a measurable transition [124]. Strict regulation of thresholds is important in applications including targeted drug delivery, sensing, and adaptive coatings, where a premature or delayed response can decrease the functionality. The threshold tuning may be obtained through module chemistry, spatial location, and local microenvironment.

**Figure 8:**
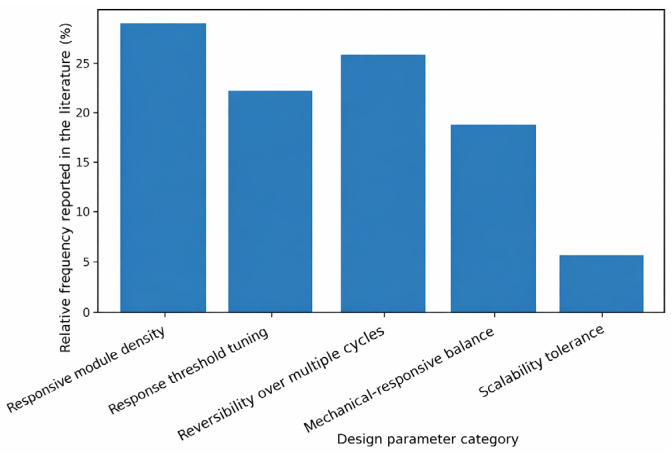
Histogram of key design parameters influencing smart polymer performance.

The other considerations of design are response reversibility and cycle stability. Smart polymers are also anticipated to operate over a long stimulus sequence without performance degradation. It needs architectures that prefer reversible interactions, but not irreversible bond cleavage, except in the case of degradation explicitly programmed. Lastly, uniformity of responses depending on architecture is becoming a measurement of its own. The homogeneous behaviour of the material leads to significant predictability of the macroscopic behaviour, whereas the heterogeneous behaviour of the material can result in either localized failure or non-uniform functioning [95]. Modular architectures are then required to be such that they are able to foster responsive elements in a manner that is controlled and statistically predictable (Figure 8).

## 8. Conclusions and Future Scope

This review offers a synthesized point of view regarding architected polymer systems by connecting the modular synthesis tactic to the stimuli-responsive design and application necessities. In contrast to previous reviews, which discuss the synthesis, the responsiveness, and applications separately, the work recognizes architectural modularity as the most significant parameter that dictates predictable structure–property relationships in the high-performance polymers. One important contribution of this review is that it has demonstrated that controlled polymerization and post-polymerization modification are synergistic in order to permit the localization of functional modules in space with a resulting high degree of specificity, sequences, and reproducible multifunctionality. This structural specificity is found to enable reversible responses to pH, temperature, light, and redox signals without affecting mechanical stability. The review also reveals that multifunctionality is not an additive effect but an interaction effect of the modules, where complex behaviour is possible, which is not possible in randomly functionalized polymers. In sum, the modular polymer architectures are also signalled as programmable platforms of future adaptive, multifunctional, and sustainable polymer systems.

In the future, the directions of future development are identified in the manuscript. The growth of multifunctionality without loss of stability is among the central concerns, and it requires further control over the hierarchical design and interactions between modules. Sustainability should be entrenched throughout the polymer conception, such as the selection of monomers, synthetic routes, and end-of-life behaviour. The predictive design tools will also be improved, which will further simplify the discovery, so that it quickly becomes possible to find the best architecture of the biomedical, energy, and environmental systems. Further development of polymer materials through the ongoing incorporation of modular synthesis, controlled responsiveness, and computational predictability can be used to provide unparalleled flexibility, environmental balance, and versatility.

## Figures and Tables

**Table 1:** Overview of key concepts in functional polymer design.

Key Concept	Description	Reference
Modular Polymer Synthesis	Enables the successive construction of polymers using diverse monomers and functional groups to customize properties.	[31]
Responsive Design	Polymers change their physical/chemical behaviour when exposed to environmental triggers such as pH, temperature, light, and redox signals.	[32]
Controlled Polymerization	Provides efficient, scalable, and sustainable synthetic processes for advanced polymer architectures.	[33]
Post-Polymerization Modification	Enhances functional diversity after backbone formation to fine-tune structure–property relationships.	[34]
Multifunctional Polymer Systems	Integration of multiple functional modules creates synergistic behaviour within a single polymer system.	[35]

**Table 2:** Architectural design strategies in modular polymer systems and their influence on adaptive functionality.

Design Parameter	Description	Role in Polymer Performance
Responsive motif selection	Choice of stimulus-sensitive functional units	Determines the type and specificity of response
Motif density and distribution	Controlled spatial arrangement of responsive groups	Enables tunable and predictable responsiveness
Stability and reversibility	Ability to undergo repeated stimulus cycles	Ensures durability and reusability
Synergistic integration	A combination of multiple responsive modules	Produces advanced behaviours such as dual-gated and multi-step responses

**Table 3:** Types of environmental stimuli and polymer responses.

Environmental Stimulus	Resulting Polymer Response	Example Functional Outcome	Reference
pH	Trigger changes in polymer conformation or behaviour	Targeted delivery or release	[143]
Temperature	Alters physical characteristics and chain mobility	Thermo-responsive coatings	[144]
Light	Activates reversible photochemical reactions	Adaptive optical materials	[145]
Redox Signals	Induces switching or chemical transitions	Self-healing or degradable systems	[146]

**Table 4:** Application module enabled by advanced functional polymers.

Application Area	How Polymers Function in This Module	Beneficial Impact	Reference
Targeted Drug Delivery	Stimuli-responsive behaviour guides site-specific release	Increased therapeutic precision	[181]
Self-Healing Materials	Responsive polymers repair themselves under environmental cues	Structural longevity	[182]
Adaptive Coatings	Polymer surfaces adjust properties with temperature/light	Smart protective surfaces	[164]
Sensing Technologies	Polymers detect environmental fluctuations via responsiveness	High-sensitivity diagnostics	[183]
Energy and Environmental Applications	Sustainable polymer architectures improve performance	Eco-friendly advanced materials	[184]

**Table 5:** Machine learning methods used for polymer structure–property prediction and representative real-world applications.

Machine Learning Method	Typical Input Features	Predicted Properties	Representative Real-World Application
Linear and Ridge Regression	Molecular descriptors, composition ratios	Mechanical modulus, glass transition temperature	Rapid screening of commodity and engineering polymers
Random Forest Models	Monomer identity, block length, topology	Thermal stability, tensile strength	Optimization of polymer blends and composites
Support Vector Machines	Chemical fingerprints, surface functionality	Solubility, permeability	Membrane materials for gas separation and filtration
Artificial Neural Networks	Sequence information, processing parameters	Nonlinear structure–property relationships	Design of stimuli-responsive drug carriers
Graph Neural Networks	Polymer graphs and connectivity	Electronic and optical properties	Flexible electronics and conductive polymer design
Bayesian Optimization	Design variables with uncertainty	Optimal property trade-offs	Accelerated discovery of multifunctional polymers

## Data Availability

No new data were created or analyzed in this study. Data sharing is not applicable to this article.

## Abbreviations

ATRP	Atom Transfer Radical Polymerization
RAFT	Reversible Addition–Fragmentation Chain Transfer Polymerization
ROMP	Ring-Opening Metathesis Polymerization
NMP	Nitroxide-Mediated Polymerization
PPM	Post-Polymerization Modification
ML	Machine Learning
DFT	Density Functional Theory
LCST	Lower-Critical Solution Temperature
UCST	Upper-Critical Solution Temperature
pH	Potential of Hydrogen
UV	Ultraviolet
GSH	Glutathione
π–π	Pi–Pi Interactions
MD	Molecular Dynamics
AI	Artificial Intelligence
RDRP	Reversible Deactivation Radical Polymerization
PEO	Poly(ethylene oxide)
Tg	Glass Transition Temperature
Mn	Number Average Molecular Weight
Mw	Weight Average Molecular Weight
Đ	Dispersity
ML-PD	Machine Learning-Based Polymer Design
GNN	Graph Neural Network
VAE	Variational Autoencoder
GAN	Generative Adversarial Network
